# Integration of rural and urban healthcare insurance schemes in China: an empirical research

**DOI:** 10.1186/1472-6963-14-142

**Published:** 2014-03-29

**Authors:** Xin Wang, Ang Zheng, Xin He, Hanghang Jiang

**Affiliations:** 1 Department of the Health Service Management, China Medical University, No.92 North Second Road, Heping District, Shenyang, Liaoning Province 110001, China(PRC; 2 China Medical University seven-year system 97 K, China Medical University, No.92 North Second Road, Heping District, Shenyang, Liaoning Province 110001, China(PRC; 3 China Medical University seven-year system 95 K, China Medical University, No.92 North Second Road, Heping District, Shenyang, Liaoning Province 110001, China(PRC; 4 China Medical University seven-year system 94 K, China Medical University, No.92 North Second Road, Heping District, Shenyang, Liaoning Province 110001, China(PRC

**Keywords:** Urban and rural healthcare insurance, Coordinated development, Empirical research

## Abstract

**Background:**

Despite the broad coverage of the healthcare insurance system in China, the imbalances in fairness, accessibility and affordability of healthcare services have hindered the universal healthcare progress. To provide better financial protection for the Chinese population, China’s new medical reform was proposed to link up urban employee basic medical insurance scheme (UEBMI), urban resident basic medical insurance scheme (URBMI), new rural cooperative medical system (NRCMS) and urban and rural medical assistance programs. In this paper, we focused on people’s expected healthcare insurance model and their willingness towards healthcare insurance integration, and we made a couple of relative policy suggestions.

**Methods:**

A questionnaire survey was conducted in four cities in China. A total of 1178 effective questionnaires were retrieved. Statistical analysis was conducted with SPSS and Excel. Chi-square test and logistic regression model were applied.

**Results and discussion:**

The payment intention and reimbursement expectation of the three groups varied with NRCMS participants the lowest and UEBMI participants the highest. In economic developed areas, rural residents had equal or even stronger payment ability than urban residents, and the overall payment intention showed a scattered trend; while in less developed areas, urban residents had a stronger payment ability than rural residents and a more concentrated payment intention was observed. The majority of participants favored the integration, with NRCMS enrollees up to 80.5%. In the logistic regression model, we found that participants from less developed areas were more likely to oppose the integration, which we conceived was mainly due to their dissatisfaction with their local healthcare insurance schemes. Also the participants with better education background tended to oppose the integration, which might be due to their fear of benefit impairment and their concern about the challenges ahead.

**Conclusion:**

Even though there are many challenges for healthcare insurance integration, it has received strong support from the mass population. However, more emphasis shall be put on equal financing and equal benefit when making further policies. As the current healthcare policies share the same design concept, principle and method, the ultimate goal of establishing a universal healthcare system is promising.

## Background

**Figure 1:**
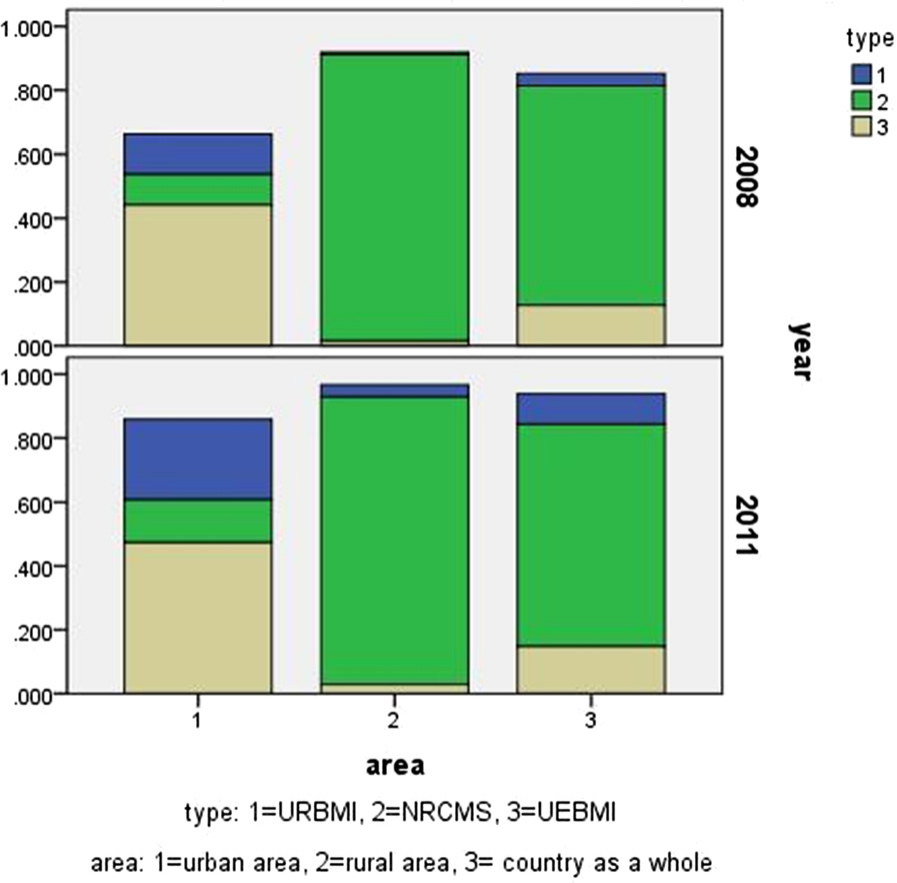
Constituents of social healthcare insurance.

China’s new medical reform established the goal of establishing the basic healthcare system and that everyone would have access to basic healthcare services covering both urban and rural residents. Generally speaking, the current healthcare insurance system in China falls into three categories, namely, the urban employee basic medical insurance scheme (UEBMI), urban resident basic medical insurance scheme (URBMI), and new rural cooperative medical system (NRCMS). In general, UEBMI stipulates that the employment-based basic health insurance scheme should cover urban employees, including employees from both public and private enterprises. Self-employed and rural industry workers are not included in UEBMI, and need to buy into the program. Retired workers are exempted from premium contributions, and their former employers should shoulder the costs of their contribution [1]. It forms risk-pooling units, which are created independently for each city or county or district [2]. URBMI, started in July 2007, was aimed to cover 240 million urban residents outside the workforce, and all urban residents would become beneficiaries by 2010 [3]. Its chief enrollees include children, college students, and migrant workers [1]. In addition to individual premiums, it is financed by central and local governments, and the funding amount per capita is approximately RMB 150 to RMB 500 ($1 US = 6.5 RMB) [2]. NRCMS is a greatly subsidized voluntary health insurance program for rural residents. It serves as a replacement for the old village-based rural health insurance program, which operates at the county level to provide a larger risk pool and economies of scale in organization and management [4]. NRCMS, started in 2003 and covering rural residents, is jointly funded by central and local governments and premium. A majority of URBMI and NRCMS benefit packages cover only inpatient care, while an increasing number of Chinese cities and counties have expanded their benefit packages to include outpatient care [5]. By the end of 2011, 97.5% of the Chinese population has been covered by the above-mentioned healthcare insurance schemes. Significant increases in the availability of healthcare insurances have been witnessed. Furthermore, a sheer reduction in the inequality of healthcare insurance between the poorest and wealthiest economic groups and regions has been observed [6,7]. Figure 1 presented the changing tendency of different population groups covered by healthcare insurance from the year 2003 to 2011.

The current healthcare insurance schemes in China have contributed to an overall improvement in its accessibility in the general population. In addition, the schemes increased outpatient and inpatient utilization and reduced delivery costs [7]. However, the imbalances in fairness, accessibility and affordability of the medical services have hindered the universal healthcare progress. The out-of-pocket payment for healthcare services remained to be high, and the financial burdens placed on individual households continued to increase, especially in the rural population, for whom the out-of-pocket payment as a share of average annual household living consumption expenditure continued to increase from 5.2% in 2000 to 8.4% in 2011 [8]. To provide a better financial protection for the vulnerable Chinese seeking essential healthcare, China’s new medical reform was proposed to link up UEBMI, URBMI, NRCMS and urban and rural medical assistance programs. Besides, the integration would also cope with the rapid urbanization that more rural residents transit to cities for employment, as the current static and separate operation mechanisms with poor interconnections would fail to meet the dynamic healthcare needs of different population groups [9].

The integrated universal healthcare insurance, though promising, faces a variety of challenges in aspects like cohesion of financing, management, payment, and service. The multiple admission standards in the current insurance system, such as census register standard (agricultural registered permanent residence and non-agricultural registered permanent residence), employment standard (practitioners with residents), and industry department standard (public officials and ordinary workers), need to be balanced to meet the healthcare needs of different population groups. In this case, mass support is a prerequisite for the successful implementation of integration. In this paper, we specifically studied people’s willingness towards the integration of healthcare systems and analyzed the factors associated with it.

## Methods

### Study sample

We conducted a household survey in four purposively selected cities, namely, Foshan, Changshu, Shenyang and Changchun. Foshan is the third biggest city in Guangdong province, with over $ 11,000 GDP per capita; Changshu, though a county-level city, is an economically developed city in Jiangsu province with over $ 20,000 GDP per capita; Shenyang is the capital city of Liaoning province, with around $ 10,000 GDP per capita; Changchun is the capital city of Jilin province, with around $ 9,000 GDP per capita (Table 1). Due to the different economic levels of the cities, some hints might be got on the status quo of healthcare insurance in different economic backgrounds. Furthermore, the healthcare insurance schemes also vary in different cities (Table 2). And the healthcare insurance administration also differs from city to city: unification of healthcare insurance administration has been achieved in Foshan, Changshu and Shenyang; while in Changchun, several departments take charge of the insurance scheme. By conducting the survey with participants with four different healthcare insurance schemes, we could better understand the most suitable model, which could be taken as a reference for the future policy making. The survey was undertaken in both urban and rural areas of the four cities, and was conducted with trained interviewers in each city to collect data from different population groups, namely, urban employees, urban residents and rural residents.

**Table 1:** Basic information of the four cities

**Indicators**	**Foshan**	**Changshu**	**Shenyang**	**Changchun**
Total population (N × 104)	726.2	150.7	822.8	758.9
Per capita GDP (RMB)	86073	123882	80532	52649
UEBMI enrollment (N)	242.4	38.9	351.2	151.0
URBMI enrollment (N)	205.8	12.2	110.8	239.2
NRCMS enrollment (N)	205.0	43.2	228.3	377.0

**Table 2:** Comparison of the health insurance schemes in the four cities

		**Changshu**	**Foshan**	**Shenyang**	**Changchun**
NRCMS	The minimum deduction line and the correspongding deduction rate	First-level and second-level designated hospitals: 100RMB; for ≦10000: 65% for (<10000)≦20000: 70% for (<20000)≦30000: 75% for >30000: 85% third-level designated hospitals: 300RMB, 60% for ≦10000: 55% for (<10000)≦20000: 60% for (<20000)≦30000: 70% for >30000: 80% outside-city designated hospitals: 500RMB, 45%	First-level designated hospitals: 300 RMB second-level designated hospitals: 500 RMB third-level designated hospitals: 700 RMB for male ≥60 years old or female ≥55 years old, the reimbursment rate is 95%; for the rest, the reimbursement rate is 90%	First-level designated hospitals: 50 RMB, 80% second-level designated hospitals: 200 RMB, 65% third-level designated hospitals: 600RMB, 40%	First-level designated hospitals: 300 RMB, 70% second-level designated hospitals: 400 RMB, 60% third-level designated hospitals: 500 RMB, 45%
	The maximum deduction line	100000 RMB	30000 RMB	30000RMB	30000RMB
URBMI	The minimum deduction line and the correspongding deduction rate	As Changshu has integrated NRCMS with URCMS, they share the same standards	First-level designated hospitals: 400 RMB, 85% second-level designated hospitals: 600 RMB, 75% third-level designated hospitals: 1200 RMB, 50%	First-level designated hospitals: for adults and aged residents:200RMB, 90% for students and juveniles: 100 RMB, 90% second-level designated hospitals: for adults and aged residents:400 RMB, 80% for students and juveniles: 200 RMB, 85% third-level designated hospitals: for adults and aged residents:600 RMB, 75% for students and juveniles: 300 RMB, 78%	First-level designated hospitals: 500 RMB second-level designated hospitals: 800 RMB third-level designated hospitals: 1200 RMB for adults and aged residents: ≦2000: 55% (>2000)≦5000: 60% (>5000)≦10000: 65% (>10000)≦30000: 70% for students and juveniles: ≦5000: 65% (>5000)≦10000: 70% (>10000)≦30000: 75% (>30000)≦50000: 80%
	The maximum deduction line		160000 RMB	For adults and aged residents:80000 RMB; for students and juveniles: 125000 RMB	For adults and aged residents:30000 RMB; for students and juveniles: 50000RMB
UEBMI	The minimum deduction line and the correspongding self-payment ratio	For in-service employees: first-level designated hospitals: 400 RMB second-level designated hospitals: 600 RMB third-level designated hospitals: 1000 RMB for retired employees: the minimum deduction line is half of the corresponding in-service employees minimum deduction line ≦10000: 12.8% (>10000)≦30000: 9.6% (<30000)≦50000: 6.4%	For in-service employees: first-level designated hospitals: 400RMB; 2% second-level designated hospitals: 600RMB; 10% third-level designated hospitals: 1200RMB; 15% for retired employees: irst-level designated hospitals: 300RMB; 0% second-level designated hospitals: 500RMB; 7% third-level designated hospitals: 1000RMB; 15%	For in-service employees: first-level designated hospitals: 300 RMB; 6% second-level designated hospitals: 500 RMB; 7% third-level designated hospitals: 800RMB; 12% for retired employees: irst-level designated hospitals: 300 RMB; 3% second-level designated hospitals: 500 RMB; 4% third-level designated hospitals: 800 RMB; 9%	The minimum deduction line is based on the average salary of Changchun last year first-level designated hospitals:9% second-level designated hospitals: 500RMB; 12% third-level designated hospitals: 800RMB; 15%
	The maximum deduction line	50000 RMB	200000 RMB (including supplemented insurance)	100000 RMB	4 times of the average salary of Changchun last year

Note: the standards above mainly refer to the in-patient care for the first time.

To interview the target population, a questionnaire was developed with three sections. Section A was designed to collect demographic information, including age, gender, education background, household economic status, recent health status, current health status, insurance enrollment status, etc. Section B was designed to study people’s payment intention and reimbursement expectation. Section C was designed to collect information about people’s willingness towards the integration of UEBMI, URBMI and NRCMS and the corresponding reasons. Informed consent was obtained from all participants following the protocol approved by Ethics Committee of China Medical University.

A total of 1200 questionnaires were distributed, with 300 in each city and 100 in every population group. We retrieved a total of 1187 questionnaires, among which 1178 were effective. The effective response rate is 98.17%.

### Measures

#### Demographic characteristics

The demographic information collected for the current study included each participant’s gender, age, type of insurance, education background and household income. The respondents were classified in accordance with their insurance type: URBMI enrollees, NRCMS enrollees and UEBMI enrollees. The annually household income was measured in Chinese Yuan as a continuous variable.

#### Payment intention

In order to measure people’s will to pay for the healthcare insurance, nine reasonable payment ranges were offered in the questionnaire, which we defined in this paper: 1 = “20 yuan or below”, 2 = “21–50 yuan”, 3 = “51–100 yuan”, 4 = “101–200 yuan”, 5 = “201–300 yuan”, 6 = “301–500 yuan”, 7 = “501–800 yuan”, 8 = “801–1000 yuan”, and 9 = “above 1000 yuan”.

#### Reimbursement expectation

To understand a reasonable reimbursement ratio, the survey was conducted with eight options, which we defined in this paper: 1 = “below 30%”, 2 = “31%–40%”, 3 = “41%–50%”, 4 = “51%–60%”, 5 = “61%–70%”, 6 = “71%–80%”, 7 = “81%–90%”, and 8 = “above 91%”.

#### Preferred healthcare insurance package

For better understanding of the respondents’ expected healthcare insurance model, three benefit packages were proposed. Package 1: pay 20 yuan every year and the reimbursement ratio is 30% to 40%; Package 2: pay 120 yuan every year and the reimbursement ratio is 50%–60%; Package 3: pay 500 yuan every year and the reimbursement ratio is 70%–80%.

### Data analysis

SPSS 17.0 and Excel were used for data analysis. Demographic characteristics of URBMI enrollees, NRCMS enrollees and UEBMI enrollees, including age, gender, education level and current health status were described (Table 3). Chi-square test was used to examine the differences between groups based on the above characteristics.

**Table 3:** Demographic characteristics of enrollees of URCMS, NRCMS and UECMS

		**URBMI**	**NRCMS**	**UEBMI**
Amount (N)		392	391	395
Gender (%)	Male	54.8	47.5	56.7
	Female	46.2	52.5	43.3
				χ^2^ = 7.345, df = 2, P = 0.025
Age (%)	20 or younger	11.0	3.1	0
	21–40	40.4	36.8	51.8
	41 or older	48.4	60.1	48.2
				χ^2^ = 72.150, df = 4, P < 0.001
Education level (%)	Elementary or lower	33.3	62.5	13.6
	Secondary to high school	35.9	24.7	26.2
	College or higher	30.8	12.8	60.2
				χ^2^ = 272.922, df = 4, P < 0.001
Health status (%)	Healthy	80.1	79.5	84.0
	Ill	19.9	20.5	16.0
				χ^2^ = 3.015, df = 2, P = 0.221

As there were three options for the integration willingness towards the three insurance schemes in the questionnaire, namely, “support”, “oppose”, “not sure”, multinomial logistic regression was applied to examine the relationship between insurance type, age, gender, education, current health status, average family annual income and integration willingness. The dependent variable was the integration willingness, where 1 = “supporting”, 2 = “opposing” and 3 = “not sure”. Independent variables included insurance type (1 = URBMI, 2 = NRCMS, 3 = UEBMI), age (1 = 20 or younger, 2 = 41 or older, 3 = 20–40), gender (1 = male, 2 = female), education (1 = elementary or lower, 2 = college or higher, 3 = secondary to high school), current health status (1 = healthy, 2 = ill), average family annual income (1 = 10000 or 10000 below, 2 = 20000 above, 3 = 10000–20000) and integration willingness. Statistical significance was defined as P ≤ 0.05.

Furthermore, payment intention, reimbursement expectation, preferred healthcare scheme, integration willingness and the corresponding reasons were described.

## Results

### Characteristics of study sample

The sample generated for analysis in our study included a total of 1178 individuals. Table 3 presented the demographic characteristics of the respondents grouped by their insurance type. The age distribution of the three groups differed significantly (χ^2^ = 72.150, df = 4, P < 0.001). UECMS group mainly consisted of the population of 20–40 years old, while the majority of URBMI and NRCMS were of over 40 years old. Education level among the three groups also differed significantly (χ^2^ = 272.922, df = 4, P < 0.001). Compared with NRCMS and URBMI enrollees (12.8% and 30.8%), more UEBMI enrollees have received college or higher education (60.2%).

### Payment intention

Table 4 showed that 25.1% of the NRCMS enrollees considered “21–50 yuan” to be the reasonable payment range, followed by 19.4% choosing “51–100 yuan” and 14.1% choosing “101–200 yuan”, presenting an overall concentrated pattern. Meanwhile, for the URBMI enrollees, “101–200 yuan” was considered the reasonable payment range by 28.6% of the respondents, followed by “51–100 yuan” (24.8%) and “21–50 yuan” (14.3%). For the UEBMI enrollees, the payment intention presented a dispersed pattern: 15.9% of “101–200 yuan”, 15.2% of “501–800 yuan”, 14.9% of “301–500 yuan”, and 14.4% of “above 1000 yuan”.

**Table 4:** Payment intention, reimbursement expectation, prefferred healthcare insurance model, integration willingness and reasons of URCMS, NRCMS and UECMS enrollees

		**URBMI (%)**	**NRCMS (%)**	**UEBMI (%)**
Payment intention	20 yuan or below	4.8	10.2	4.8
	21–50 yuan	14.3	25.1	8.4
	51–100 yuan	24.8	19.4	9.9
	101–200 yuan	28.6	14.1	15.9
	201–300 yuan	10.7	9.5	7.4
	301–500 yuan	9.4	5.1	14.9
	501–800 yuan	3.6	4.3	15.2
	801–1000 yuan	1.8	7.4	9.1
	above 1000 yuan	2.0	4.9	14.4
	Total	100.0	100.0	100.0
Reimbursement expectation	30% or below	0.0	3.6	0.0
	31%–40%	3.6	4.6	0.0
	41%–50%	4.1	5.6	1.5
	51%–60%	5.1	10.7	1.8
	61%–70%	6.1	5.1	2.3
	71%–80%	31.4	27.4	18.7
	81%–90%	30.1	25.6	38.2
	above 90%	19.6	17.4	37.5
	Total	100.0	100.0	100.0
Preferred healthcare insurance package	Package 1	11.7	19.2	5.6
	Package 2	57.9	47.1	36.7
	Package 3	30.4	33.7	57.7
	Total	100.0	100.0	100.0
Integration willingness	Support	69.6	80.5	57.7
	Oppose	18.9	8.2	30.6
	Not sure	11.5	11.3	11.7
	Total	100.0	100.0	100.0
Reasons of supporting healthcare insurance integration	It would achieve equal access to healthcare services.	45.0	65.7	64.0
	There would be more options of hospital for participants.	48.7	40.6	27.1
	It would reduce the healthcare gap between urban and rural areas.	31.5	38.4	38.5
	Participants would enjoy better healthcare services.	35.8	27.3	47.9
	It would improve the overall risk resistance ability of insuran scheme.	12.4	10.1	25.8
	It would facilitate the labour flow between urban and rural areas.	6.2	8.8	17.1
	It would reduce the administration costs.	7.6	3.4	14.4
	Others.	1.8	2.2	1.7
Reasons of opposing healthcare insurance integration	The payment and treatment standards of the systems are different.	36.4	46.8	31.4
	Administration of insurance may fall behind after integration.	39.1	28.1	32.2
	Urban and rural participants have different healthcare needs.	31.0	46.8	21.4
	Some pilot areas should be implemented before implementation on a large scale.	28.3	21.8	25.6
	Some people may take advantage of the integrated insurance scheme	33.7	34.3	17.3
	The conditions for integration are not yet ripe.	29.7	25.0	13.0
	Others.	25.6	40.6	18.1

**Figure 2:**
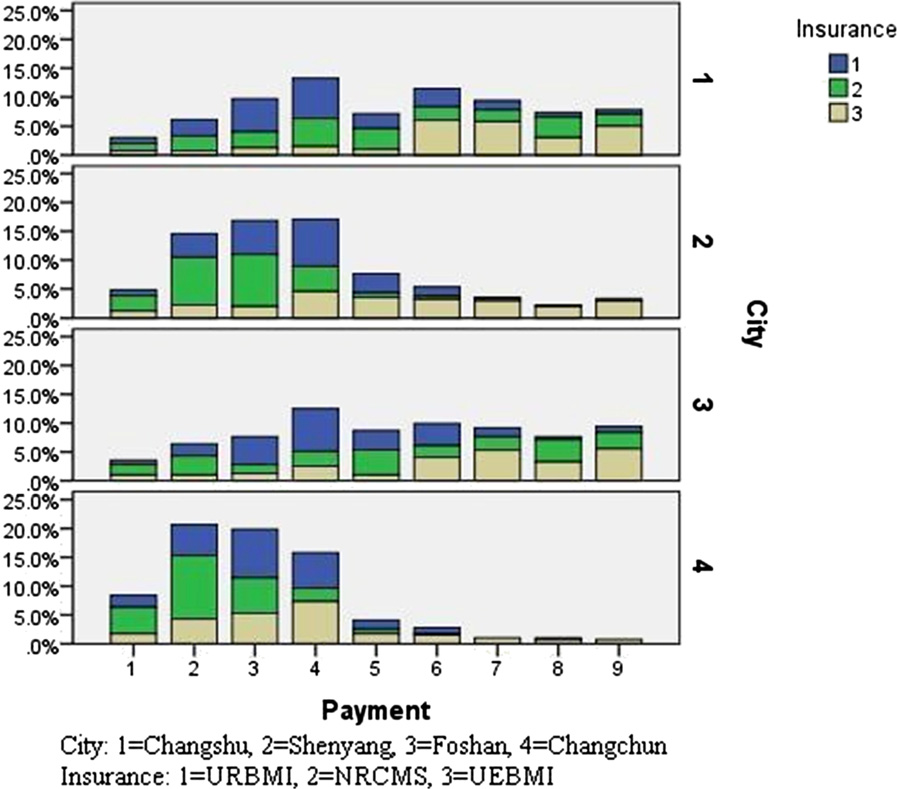
Payment intention of URBMI, NRCMS, UEBMI enrollees by city.

When analyzed by regions, in economic developed areas like Foshan and Changshu, rural residents had equal or even stronger payment ability than urban residents, and the overall payment intention presented a scattered trend, as was shown in Figure 2. While in less economically developed areas like Changchun and Shenyang, urban residents had a stronger payment ability than rural residents and more concentrated payment intention could be observed.

### Reimbursement expectation

Among the three groups of population, NRCMS enrollees presented the lowest reimbursement expectation: 27.4% chose “71%–80%”, 25.6% chose “81%–90%”, 10.7% chose “51%–60%”. A more concentrated pattern could be observed in URBMI enrollees: 31.4% chose “71%–80%”, 30.1% chose “81%–90%”, 19.6% chose “above 90%”. And UEBMI enrollees held the highest reimbursement expectation: 38.2% chose “81%–90%”, 37.5% chose “above 90%”, 18.7% chose “71%–80%”.

**Figure 3:**
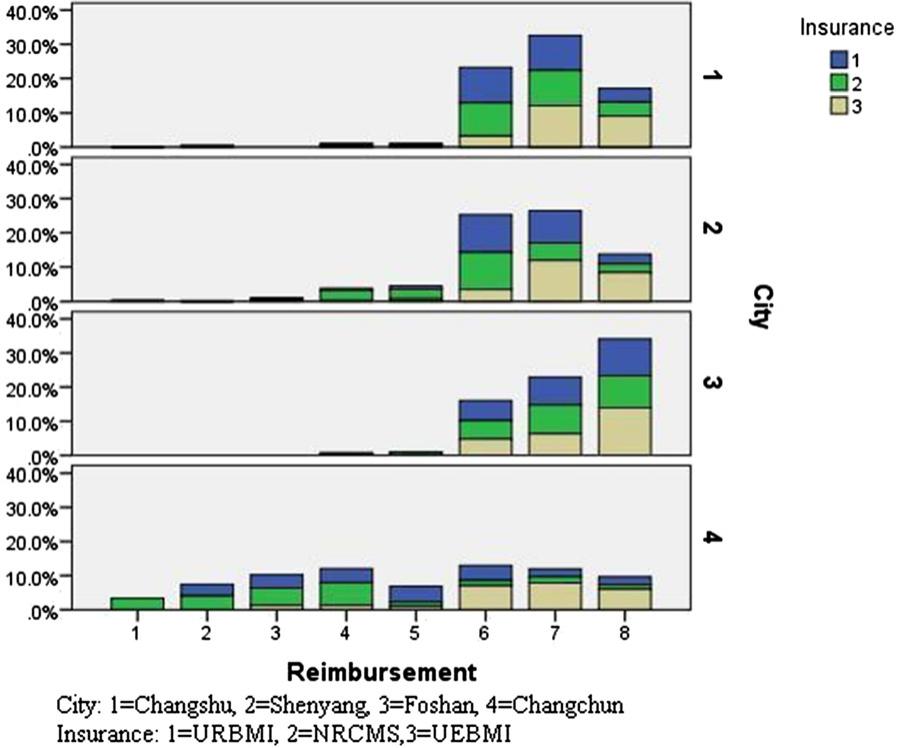
Reimbursement expectation of URBMI, NRCMS, UEBMI enrollees by city.

As was shown in Figure 3, urban employees, urban residents and rural residents in Foshan, compared with these in the other three cities, had the highest and similar reimbursement expectations. In addition, in Changshu, urban employees had higher reimbursement expectations, while urban and rural residents shared similar and lower expectations, which is consistent with the local policy of integrating rural resident healthcare insurance with urban resident healthcare insurance. In Shenyang, reimbursement expectations of the three groups were mainly distributed at three intervals. In Changchun, generally low and scattered reimbursement expectations have been witnessed: rural residents held the lowest reimbursement expectations and urban employees held the highest reimbursement expectations.

### Preferred healthcare insurance package

Given the three benefit packages, with the best healthcare treatment in Package 3, followed by Package 2 and Package 1, a majority of URBMI (57.9%) and NRCMS (47.1%) enrollees preferred Package 2, and 36.7% of UECMS enrollees chose it. While the majority of UECMS enrollees took a fancy to Package 3, taking up 57.7%.

**Figure 4:**
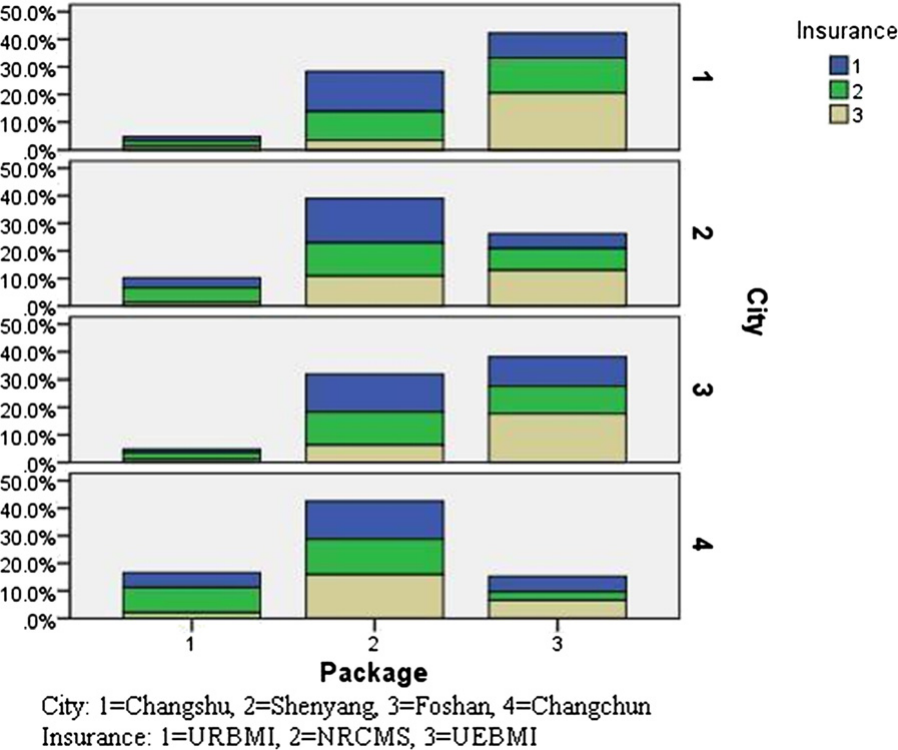
Preferred healthcare benefit package of URBMI, NRCMS, UEBMI enrollees by city.

In Figure 4, the majority of Foshan and Changshu participants preferred Package 3, while the majority of Shenyang and Changchun participants preferred Package 2. Furthermore, in the same area, people’s preference differed within the same group, regardless of the economic level.

### Integration willingness and the corresponding reasons

Among the 1178 respondents, 816 individuals were in support of the integration of NRCMS, URBMI and UEBMI, accounting for 69.3%. The majority of URCMS, NRCMS and UEBMI enrollees were in favor of the integration, taking up 69.6%, 80.5% and 57.7%, respectively. However, 30.6% of the UEBMI enrollees, 8.2% of the NRCMS enrollees and 18.9% of the URBMI enrollees opposed the integration.

The top reason for the integration was that it would achieve equal access to healthcare services for both rural and urban participants. Also a great number of supporters believed that they would have more choices of hospitals after integration. Other main reasons were that it would narrow the healthcare gap between urban and rural areas, and that the participants would enjoy better healthcare services.

The most common reason against the integration was that the payment ranges and benefit packages in the current insurance schemes were quite different, making it hard and unsuitable to integrate. Some participants also worried that the administration might fall behind after integration and the healthcare needs of urban and rural participants were different, as details described in Table 4.

### Factors associated with integration willingness

In the multinomial logistic regression model (Table 5), a participant was more likely to oppose the integration in less economically developed areas, which, in this case, was Changchun. It indicated that the economic background of the coordinated area might affect people’s willingness towards integration. Furthermore, participants’ education background might also play a role in the integration willingness. In our study, surprisingly, we found people with higher education levels were more likely to oppose the integration.

**Table 5:** Results of logistic regression on factors affecting integretion willingness

	**Support**			**Oppose**		
	**B**	**Odds ratio (95% CI)**	**P**	**B**	**Odds ratio (95% CI)**	**P**
Male	0.039	1.040 (0.722–1.497)	0.834	0.266	1.305 (0.851–2.003)	0.223
Age <20	–0.391	0.676 (0.283–1.618)	0.380	0.333	1.394 (0.553–3.515)	0.481
Age >40	0.007	1.007 (0.691–1.467)	0.972	–0.348	0.706 (0.454–1.100)	0.124
URBMI	0.202	1.224 (0.783–1.914)	0.376	–0.470	0.625 (0.378–1.033)	0.067
NRCMS	0.368	1.444 (0.924–2.259)	0.107	–1.286	0.276 (0.157–0.488)	P < 0.001
Elementary school or lower	0.083	1.087 (0.697–1.695)	0.713	–0.835	0.434 (0.230–0.817)	0.010
College or higher	–0.286	0.752 (0.472–1.197)	0.229	1.091	2.979 (1.738–5.104)	P < 0.001
Household annual income per capita <10000 RMB	0.355	1.426 (0.912–2.230)	0.119	–0.956	0.384 (0.195–0.759)	0.006
Household annual income per capita ≧20000 RMB	–0.257	0.774 (0.497–1.205)	0.256	1.017	2.764 (1.694–4.509)	P < 0.001
Foshan	–0.314	0.730 (0.491–1.244)	0.248	–0.041	0.960 (0.515–1.789)	0.960
Shenyang	–0.097	0.907 (0.526–1.564)	0.726	–0.185	0.831 (0.434–1.590)	0.831
Changchun	–0.573	0.564 (0.335–0.948)	0.031	–0.031	0.970 (0.531–1.770)	0.970

Integration willingness (1 = support, 2 = oppose, 3 = not sure).

## Discussion

China’s healthcare insurance system has multiple admission standards and multiple divisions, which go against the status quo of flow of personnel, and may jeopardize the robustness and sustainability of healthcare insurance financing through the allocating the risk to public, directly doing harm to the efficiency of operation [10]. Take the example of NRCMS, which is designed exclusively for rural people according to their hukou [4], the enrollees are expected to seek medical services in the designated hospitals, most of which are located within the home county, which is impractical for the migrant job seekers far away from home, limiting their NRCMS benefits. Furthermore, the migrants are also facing the lack of accessibility to healthcare insurance in cities, as most of them are not qualified for UEBMI and URBMI [11,12].

In 2009, a new round of healthcare system reform has been initiated, aiming to ensure that the State plays a critical role in guaranteeing universal coverage of essential healthcare and providing efficient, convenient and affordable basic healthcare services [13]. Four aspects of healthcare system covering both urban and rural residents are involved in the reform: health insurance schemes, national essential drug systems, clinical service systems and the public health/preventive service systems. Hitherto, the healthcare insurance schemes in China have achieved phasic success. Despite the increasing coverage of population, unfairness, inequality and unaffordability of the healthcare services have hindered the universal healthcare progress, which might also affect people’s enthusiasm for participating in the healthcare insurances. It should be noted that their lack of participation will not only lead to adverse selection but also to considerably higher administrative costs [14].

### Healthcare insurance expectations, healthcare insurance integration willingness and associated factors

To promote the participation in healthcare insurances and to smoothly integrate UEBMI, URBMI and NRCMS, the voices of mass population should be heard. Our data revealed that the expected payment range of urban employees presented a dispersed trend, while that of urban residents and rural residents presented a concentrated pattern (Figure 2). This could be explained by the fact that the insurance payment of urban employees is proportional to their salaries, while for urban and rural residents, the quota payment, which sets a payment standard for enrollees, limits their choices. The UEBMI guideline established by State Council suggests premium contributions by employers and employees be set at 6% and 2% of an employee’s salary, respectively [15]. While according to a URBMI survey conducted by State Council, the average per capita financing level of the pilot cities in 2007 reached 236 RMB for adults and 97 RMB for minors. About 36% and 50% of these amounts, respectively, were contributed by government subsidies, indicating that the majority of funding was obtained from individual contributions [7]. The total government subsidies for NRCMS enrollees were 80 RMB per rural resident in 2009, of which central and local government each contributed 40 RMB. Meanwhile, individual contributions rose to 20 RMB per enrollee and the average financing level increased to 100 RMB per enrollee in 2009 [16]. As the healthcare insurance systems of URBMI and NRCMS have been integrated in some areas, the participants shared the same reimbursement expectations (Figure 3). In addition, as indicated in our study, people in economically developed areas showed higher payment intention, higher reimbursement expectation as well as better insurance benefit package. In addition, according to the 2008 National Health Service Survey [17], income level was a major determinant of health outcomes. Therefore, the economic status of participants from different areas and different population groups should be taken as a reference when setting the payment standard and benefit package.

As noted in Table 4, rural residents were the most supportive of healthcare insurance integration, with the most common reason of achieving equal access to healthcare services. On the other hand, people who strongly opposed the integration mainly held that the gaps in the insurance coverage and healthcare benefits across different schemes remained to be so wide that it was unwise to integrate them at this moment. Despite the increasing coverage of healthcare insurance nationally [18], the access to healthcare services remained to be uneven, and the integration would not only promote the benefits for rural residents, but will also provide much convenience for rural migrants seeking medical care in cities. Jian and his colleagues have reported that even though rural Chinese with chronic disease could more easily start inpatient treatment in 2008 than they could in 2003, they were more than twice as likely to drop out of treatment as were Chinese in urban areas due to the higher hospital copayments required under insurance coverage for rural citizens [19]. Therefore, in the process of coordinating urban and rural healthcare insurance schemes, the economic background should not be neglected. The logistic regression model in Table 5 indicated that participants in Changchun were more likely to oppose the integration than the participants in the other three cities, reflecting that the enrollees in Changchun were not satisfied with the current insurance schemes. Also we found that people with better education background tended to oppose the integration, which might seem contradictory to the common sense. We proposed two reasons for it: on one hand, as no complete integration policy has been implemented yet, they might concern that the integration would drag down their healthcare benefits; on the other hand, they might be better aware of the underlying challenges of integration, involving limited financial risk pooling, inefficient purchasing and provider incentives, etc. In this case, a proper balance of benefits among each group is crucial in improving the healthcare scheme.

### Policy implication

According to the feedback from the enrollees, we have come up with the following suggestions to improve the healthcare insurance policy.

Setting up a multi-grade payment and treatment standard: For developed areas, high subsidies are provided to ensure that urban and rural residents receive improved or even equivalent healthcare treatment as urban employees (such as Changshu and Foshan). While for most parts of China, where the income of urban and rural residents differ greatly and the local fund is limited, a proper fund-raising mechanism with multiple standards is needed in the transitional stage, so that participants could choose the grade according to their own economic background.

Constructing an integrated healthcare insurance information system: For areas that are immature to integrate URBMI and NRCMS, instead of leaping to the integration, a unified information platform shall be established as the first move towards the integration.

Unifying the administration: To improve the poor interconnections due to the separate healthcare administrative institutions, a unified administration will not only facilitate the coordination of healthcare insurances, but will also alleviate the financial pressure of operation. The unified policies in Foshan, Changshu and Shenyang have set successful examples for broader implementation.

Establishing a proper transferring mechanism: With the market economic development and agricultural market reforms, many rural workers transit to cities for employment, most of whom receive little NRCMS reimbursement [9]. A transferring mechanism that links with the original system could well improve the situation. But it requires a cohesive mechanism among different systems, and solving problems like how to convert payment age limitations and how to compensate for the medical funding of the local area.

Improving the coordination level: Currently, the healthcare insurance coordination remains at various levels, including county level, municipal level, and provincial level. This leads to the poor risk-resisting ability. While by improving the planning on a higher level, we could have a stronger ability to resist risks, thus increasing enrollees’ confidence in healthcare insurances.

Setting up a stable fund-raising and financial subsidy mechanism: In spite of the increasing funding level, the financing mechanism is not well regulated. We propose that the payment standard of rural residents shall be set in accordance with their net income, while that of urban residents shall be set in accordance with their disposable income, and financial subsidies from the government shall be determined partially by residents’ hospitalization cost.

### Limitations

The study has several limitations. Firstly, the selection of the respondents focused on certain communities in the four cities, and the results may not be generalizable to the entire country due to differences among areas, and could not fully represent the three groups of population, namely, rural residents, urban residents and urban employees. Secondly, the differences of the same insurance scheme across cities were not considered when we studied the factors influencing participants’ willingness towards healthcare insurance integration. Thirdly, some data in out study, like household annual income per capita, were collected on the basis of personal recall and could be prone to measurement errors.

## Conclusion

This study featured enrollees’ willingness towards integration of various healthcare insurances and the relevant factors. In spite of the expanding coverage of healthcare insurance, the imbalance in fairness, accessibility and affordability of the medical services have hindered the universal healthcare progress. Fear of impaired healthcare benefit and the differences of URBMI, NRCMS, UEBMI in aspects like administration, payment standard, benefit package, etc. were found to be the main reasons for opposing healthcare insurance integration. Therefore, the key to coordinating urban and rural healthcare insurance lies in equal financing and equal benefit. Further studies shall be conducted on the role of healthcare providers in terms of the integrated healthcare scheme. Policy makers shall find a balance between health providers and participants: enough compensation shall be distributed to health providers, mostly to public healthcare institutions, and also affordability to healthcare service shall be ensured [20]. Combining the suggestions above, despite the challenges ahead, a new integrated healthcare scheme is promising and could be applied in the near future.

## Competing interests

The authors declare that they have no competing interests.

## Authors’ contributions

XW and AZ participated in conduct of the study, data analysis and manuscript drafting. XW participated in the project design, co-ordination and conduct of the study. XH and XW participated in the project design, conduct of the study, and revision of drafts of the manuscript. All authors read and approved the final manuscript.

## Authors’ information

Xin Wang, female, Department of the Health Service Management, China Medical University, Associate Professor of Health Economics, PhD. Executive Director of China Health Economic Association. Director of China Soft Science Association, Master Tutor, Major Research on: Health Economic and Policy.

Ang Zheng, male, Master, 97 K Seven-years system, China Medical University.

Xin He, female, Master, 95 K Seven-years system, China Medical University.

Hanghang Jiang, female, Master, 94 K Seven-years system, China Medical University.

## Pre-publication history

The pre-publication history for this paper can be accessed here:

http://www.biomedcentral.com/1472-6963/14/142/prepub
